# Identification of powdery mildew resistance in wild grapevine (*Vitis vinifera* subsp. *sylvestris* Gmel Hegi) from Croatia and Bosnia and Herzegovina

**DOI:** 10.1038/s41598-022-06037-6

**Published:** 2022-02-08

**Authors:** Katarina Lukšić, Goran Zdunić, Katarina Hančević, Maja Žulj Mihaljević, Ana Mucalo, Erika Maul, Summaira Riaz, Ivan Pejić

**Affiliations:** 1 Institute for Adriatic Crops and Karst Reclamation, Put Duilova 11, 21 000 Split, Croatia; 2 University of Zagreb, Faculty of Agriculture, Svetošimunska cesta 25, 10 000 Zagreb, Croatia; 3 Julius Kühn-Institute, Federal Research Centre for Cultivated Plants, Institute for Grapevine Breeding Geilweilerhof, 76833 Siebeldingen, Germany; 4 Department of Viticulture and Enology, University of California, Davis, CA 95616 USA; 5 Centre of Excellence for Biodiversity and Molecular Plant Breeding, 10 000 Zagreb, Croatia

**Keywords:** Plant breeding, Agricultural genetics

## Abstract

Wild grapevine (*Vitis vinifera* subsp. *sylvestris*) is widely recognized as an important source of resistance or tolerance genes for diseases and environmental stresses. Recent studies revealed partial resistance to powdery mildew (*Erysiphe necator,* PM) in *V. sylvestris* from Central Asia. Here, we report resistance to PM of *V. sylvestris* collected from different regions of Croatia and in seedling populations established from in situ* V. sylvestris* accessions. Ninety-one in situ individuals and 67 *V. sylvestris* seedlings were evaluated for PM resistance according to OIV 455 descriptor. Three SSR markers (SC47-18, SC8-071-0014, and UDV-124) linked to PM resistance locus *Ren1* were used to decipher allelic structure. Nine seedlings showed resistance in in vivo evaluations while leaf disk assays revealed three PM-resistant accessions. One *V. vinifera* cultivar used as a control for PM evaluations also showed high phenotypic resistance. Based on the presence of one or two resistance alleles that are linked to the *Ren1* locus, 32 resistant seedlings and 41 resistant in situ genotypes were identified in the investigated set. Eight seedlings showed consistent phenotypic PM resistance, of which seven carried one or two alleles at the tested markers. This study provides the first evidence of PM resistance present within the eastern Adriatic *V. sylvestris* germplasm.

## Introduction

Powdery mildew (*Erysiphe necator*) is an economically important fungal disease of grapevine. It has been a continuous problem since its introduction from North America to Europe around 1845. In < 10 years, *E. necator* became a problem in vineyards throughout the Mediterranean^1^. Shortly after the onset of this disease in Europe, inorganic fungicides and sulfur were used to control powdery mildew (PM). There is a risk of the fungus developing resistance to fungicides^2^. Chemical protection is applied ~ 10 times during the growing season, making viticulture one of the largest fungicide consumers worldwide^3^. Fungicides can also cause undesirable characteristics, such as off-flavors in wine^4^. Modern researchers are developing revolutionary methods to control PM, including exposure of the fungus to UV light at night, when the fungal defense system ‘turns off’^5^. Another solution comes from the grapevine genome itself. Twelve loci from diverse grapevine species originating in North America, Central Asia and China carry genes for plant defense against PM^6^. Loci such as *Ren4* and *Ren6* (Resistance to *Erysiphe necator*) provide complete resistance, not allowing the fungus to proliferate^7^.

European grapevine (*Vitis vinifera* subsp. *vinifera;* hereafter called *V. vinifera*) is susceptible to powdery mildew, generally showing no resistance^8^. However, strong natural resistance to PM was first identified in the Central Asian *V. vinifera* cultivar (cv.) ‘Kishmish vatkana’^9^, which carries the major locus *Ren1* on chromosome 13. The *Ren1* locus has many advantages in breeding programs and mediates partial resistance to PM^6^, similar to loci *Ren3* and *Ren9*^10^.

Research on PM resistance was recently broadened by including the wild Eurasian grapevine (*Vitis vinifera* subsp. *sylvestris* Gmel Hegi; hereafter called *V. sylvestris*) into disease evaluations. *V. sylvestris* usually grows in habitats isolated from human impact. It is mostly susceptible to PM^11,12^, but considerably less affected by the disease than *V. vinifera*^11,13^ indicating some level of tolerance to PM^11^. *V. sylvestris* populations in northern Spain were susceptible to PM^12^, as were vines growing in six river basins in southern Spain^14^, with varying infection levels of different parasitic species in vines from the same location.

Recent identification of *Ren1* in two *V. sylvestris* accessions from Central Asia revealed that PM resistance is located at the same genetic position on chromosome 13 as in cv. ’Kishmish vatkana’^15^. Additionally, a few dozen PM-resistant *V. sylvestris* accessions from Central Asia were identified as carriers of new resistance-linked alleles at five microsatellite (SSR) markers associated with the *Ren1* locus^7^. The presence of *Ren1* in *V. sylvestris* is very intriguing for the grapevine breeding community. The genetic resources of *V. sylvestris* in European populations represent important sources of genetic variability that are worth preserving. However, no research has investigated the presence of resistance-linked alleles at the *Ren1* locus.

*V. sylvestris* has been referenced in Croatian literature historically^16,17^. Recently, Croatian *V. sylvestris* was systematically identified and characterized^18–20^ as belonging to five natural populations in humid Mediterranean forests and in sub-Mediterranean ecosystems^19^. Prospecting at natural sites is ongoing and recently two additional populations were identified (data not published). However, these studies did not evaluate resistance to fungal disease among the populations.

To bridge that gap, the present work (i) evaluates PM resistance of *V. sylvestris* accessions from the eastern Adriatic region (Croatia and Bosnia and Herzegovina) using simple sequence repeat (SSR) markers linked to the powdery mildew resistance locus *Ren1*, and (ii) performs a detailed phenotypic evaluation of visible powdery mildew symptoms on *V. sylvestris* seedlings. This is the first report of PM evaluations in *V. sylvestris* collected in Croatia and Bosnia and Herzegovina and identifies germplasm that could be used for grapevine breeding.

## Results

### Disease evaluations

The inventory of *V. sylvestris* in their natural habitats found very few vines with powdery mildew (PM) symptoms. Only ~ 7% of individuals developed PM symptoms, mostly from the Krka National Park population. Detailed evaluation for PM resistance continued on 67 V*. sylvestris* seedlings grown in pots in an ex-situ seedling collection.

Seedlings were previously established from seeds of five female individuals during inventory work aiming at gene conservation. Each seedling was represented by one biological replicate due to juvenile growth phase and lower plant vigour in a shaded collection.

**Figure 1:**
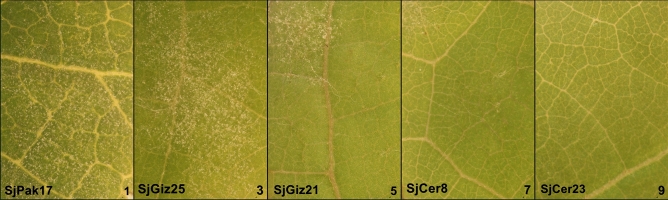
Grapevine powdery mildew (*E. necator*) mycelium development on *V. sylvestris* leaves. Powdery mildew spores were inoculated onto the top (adaxial) surface of leaf disks of *V. sylvestris* seedlings. Each figure represents fungal growth on a single leaf disk of five different genotypes according to the OIV 455 scale from 1 to 9 (left to right), respectively. The PM-susceptible seedlings received a lower scale number, while resistant seedlings received a higher number.

Visual PM symptoms on each accession were rated according to the OIV 455 descriptor using a five-class scale (1–9), for both in vivo observations of the collection and in vitro leaf disk testing (Fig. 1).

**Figure 2:**
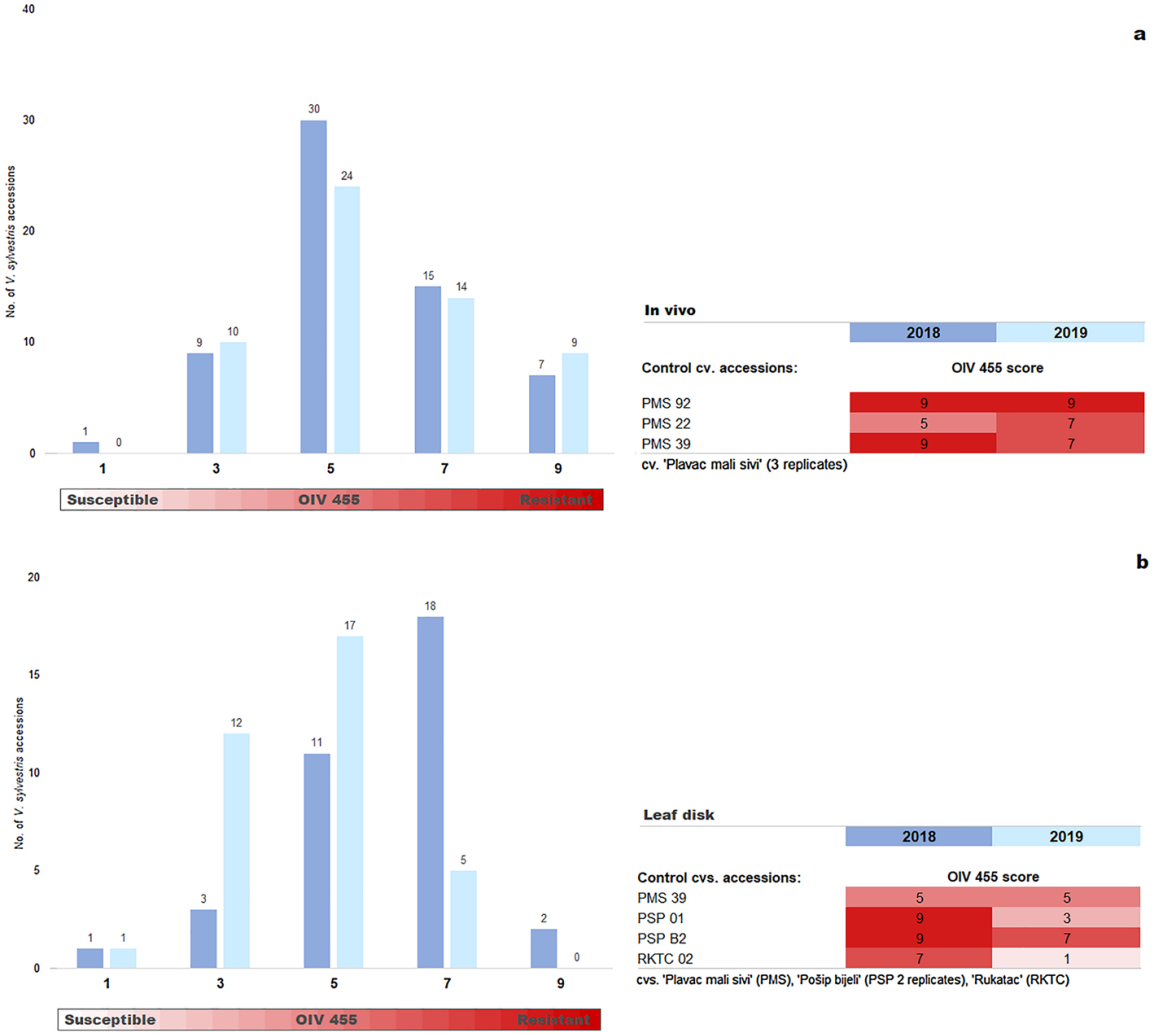
Resistance of *V. sylvestris* seedlings to grapevine powdery mildew (*E. necator*) based on OIV 455 descriptor five-class scale (1–9) and two phenotypic approaches: (**a)** In vivo evaluation in 2018 encompassed 62 accessions and 57 accessions in 2019. *V. vinifera* cv. ‘Plavac mali sivi’ (PMS) was used as control. (**b)** Leaf disk assay on a subset of 35 *V. sylvestris* accessions in 2018 and 2019. Three *V. vinifera* cvs. were used as controls. Each chart column in **(a,b)** shows the number of different accessions sharing the same OIV score (level of resistance) in the year of evaluation. Unless otherwise indicated, all accessions were represented by a single biological replicate.

Two-year in vivo evaluation of PM on entire seedling plants was possible for 62 and 57 seedlings out of 67 seedlings in 2018 and 2019, respectively (Fig. 2a). Failing accessions either dried up or were too small at the time of observation. Nearly half of the seedlings analyzed in 2018 (30 accessions) showed intermediate PM resistance (score 5). Twenty-two accessions were resistant: 15 resistant and seven very resistant (scores 7 and 9, respectively). Nine accessions were susceptible and one was very susceptible to PM (scores 3 and 1, respectively). Similar trends were observed in 2019. In both years, intermediate resistance was confirmed in 12 accessions, resistance in nine and susceptibility in four (Table 1). Three control accessions of *V. vinifera* cultivar ‘Plavac mali sivi’ showed resistance to high resistance, except that accession PMS22 had intermediate resistance in 2018 (Fig. 2a).

**Table 1:**
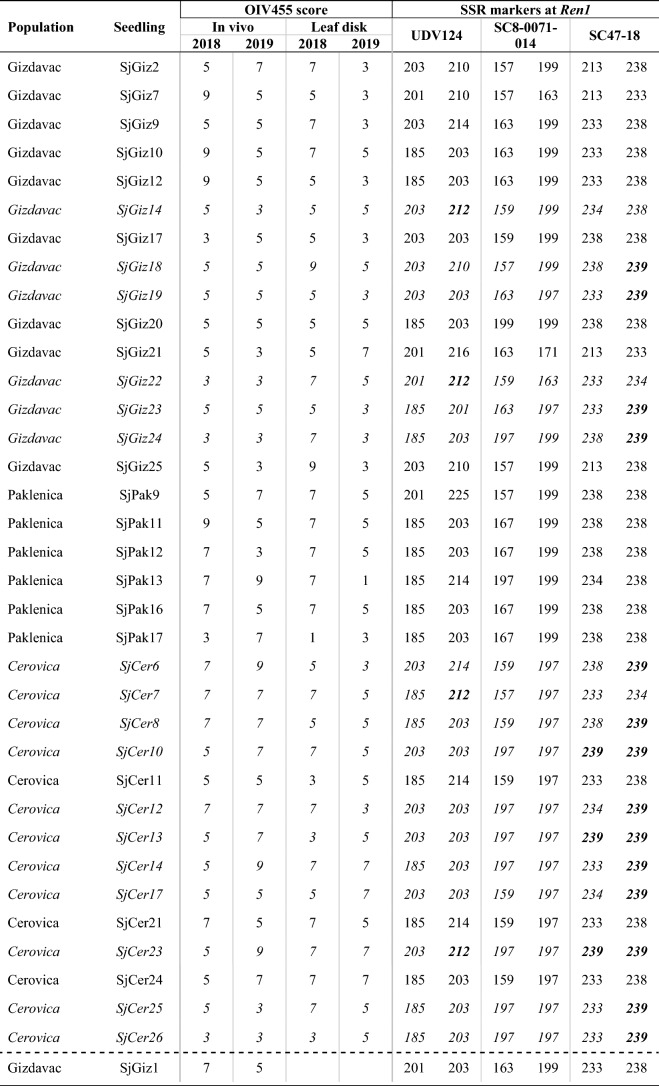

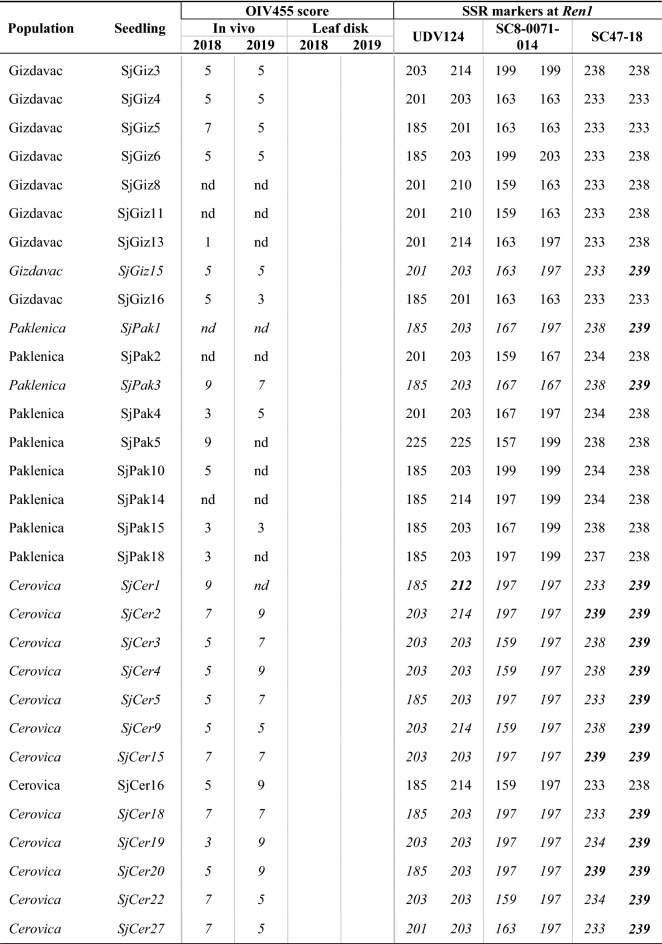
Genetic polymorphism of 67 *V. sylvestris* seedlings at three SSR markers linked to the *Ren1* locus on chromosome 13 and phenotypic powdery mildew results obtained from in vivo and leaf disk methods based on the OIV 455 descriptor scale from 1 (susceptible) to 9 (resistant) in 2018 and 2019.

Dashed line separates seedlings based on phenotypic method used for their disease evaluation. For 10 seedlings, phenotypic results were not determined or not complete (‘nd’) as accessions dried or were too small for evaluation. Rows in italics individuals carrying R-alleles. Allele 239 at marker SC47-18 (in bold) was linked to resistance in *V. sylvestris.* Allele 212 at marker UDV124 (in bold) was linked to resistance in *V. vinifera*^7^.

A subset of 35 seedlings with the best fitness in 2018 and 2019 was chosen for the leaf disk assay. Three *V. vinifera* cvs. were used as controls (Fig. 2b). The majority of seedlings (20 accessions) in 2018 showed PM resistance: 18 were resistant and two very resistant. Intermediate resistance was found in 11 accessions and four were susceptible. In 2019, only five accessions of the same subset were resistant, 17 accessions had intermediate resistance, while 13 were susceptible. Three seedlings (SjCer14, SjCer23 and SjCer24) had leaf disk PM resistance in both years. Three seedlings had intermediate resistance and one was susceptible in both years (Table 1). Control cv. 'Plavac mali sivi' (PMS 39) showed intermediate resistance, 'Pošip bijeli' (PSP 01 and PSP B2) displayed very high resistance in 2018, but were susceptible and resistant, respectively, in 2019. 'Rukatac' (RKTC 02) was rated highly resistant in 2018 and very susceptible in 2019.

### Genetic polymorphism at the *Ren1* locus with reference to phenotypic disease evaluation

Genetic SSR data based on three markers: SC47-18, SC8-0071-14 and UDV124, were adapted to match alleles with previous studies^7,15^. Thirty-two out of 67 seedlings had the R-allele at one or two SSR markers (Table 1). R-allele 239 at marker SC47-18 was the most frequent (29 seedlings). Five seedlings had R-allele 212 at UDV124. Two seedlings had both 239 and 212 R-alleles.

Seedlings from the Cerovica population showed the greatest overall phenotypic resistance (mean value 6), while seedlings from the Gizdavac population had the least overall resistance (4.97), despite relatively high R-allele variability.

None of the seedlings showed full resistance, evaluated using two phenotypic approaches, over both years (Table 1). However, three seedlings were resistant when analyzed in vivo and in one year of leaf disk assay: SjPak13 (no R-alleles), SjCer7 (allele 212), and SjCer12 (allele 239). Three Cerovica seedlings, SjCer14, SjCer23, and SjCer24, showed resistance in leaf disk assays and in one year of in vivo. Seedling SjCer23 had two R-alleles (239 and 212), SjCer14 one (239), and SjCer24 had none of the R-alleles.

### PM resistance of in situ* V. sylvestris* genotypes

Ninety-one *V. sylvestris* individuals from natural habitats (in situ) were analyzed at three SSR markers (Table 2). Forty-one individuals carried alleles associated with PM resistance. All these individuals had R-allele 239 at SC47-18 except one (Im4), which had R-allele 246 at the same marker. One accession (Luk8) contained both alleles: 239 and 246. The Paklenica population had the most individuals carrying R-alleles (13), followed by Psunj (12) and Lukovdol (8). Populations Imotski (4), Krka (2) and Gizdavac (1) had the fewest individuals with R-alleles.

**Table 2:** Genetic polymorphism of 91 in situ-tested *V. sylvestris* individuals at three SSR markers linked to the *Ren1* locus on chromosome 13.

Population	Genotype	SSR markers at *Ren1*					
		UDV124		SC8-0071-014		SC47-18	
Paklenica	Pak1	203	225	157	159	234	238
Paklenica	Pak2	201	203	167	167	238	238
Paklenica	Pak3	201	225	157	167	238	238
Paklenica	Pak5	201	225	157	167	228	238
Paklenica	Pak6	185	201	167	197	234	234
*Paklenica*	*Pak7*	*185*	*201*	*197*	*197*	***239***	***239***
*Paklenica*	*Pak8*	*201*	*203*	*167*	*199*	*234*	***239***
*Paklenica*	*Pak9*	*185*	*201*	*163*	*197*	*233*	***239***
Paklenica	Pak10	185	225	167	167	228	238
*Paklenica*	*Pak11*	*203*	*203*	*167*	*197*	*238*	***239***
*Paklenica*	*Pak12*	*203*	*225*	*157*	*167*	*238*	***239***
*Paklenica*	*Pak13*	*185*	*225*	*157*	*197*	*238*	***239***
Paklenica	Pak14	183	225	157	157	238	238
*Paklenica*	*Pak15*	*201*	*225*	*197*	*199*	*238*	***239***
Paklenica	Pak16	201	201	159	199	234	238
Paklenica	Pak17	201	225	159	203	238	238
Paklenica	Pak18	185	201	197	199	234	238
Paklenica	Pak19	183	185	157	167	238	244
*Paklenica*	*Pak20*	*183*	*201*	*157*	*197*	***239***	*244*
Paklenica	Pak21	201	203	167	199	238	238
*Paklenica*	*Pak22*	*185*	*201*	*159*	*197*	*238*	***239***
*Paklenica*	*Pak23*	*185*	*201*	*159*	*197*	*238*	***239***
*Paklenica*	*Pak24*	*185*	*203*	*163*	*197*	*233*	***239***
*Paklenica*	*Pak25*	*183*	*185*	*157*	*197*	***239***	*244*
Paklenica	Pak26	185	185	161	197	220	234
Paklenica	Pak27	183	201	157	167	233	238
Paklenica	Pak28	185	203	167	197	234	238
Paklenica	Pak29	185	214	199	199	238	238
*Paklenica*	*Pak30*	*201*	*225*	*163*	*197*	*233*	***239***
Paklenica	Pak32	201	203	167	199	238	238
Paklenica	Pak33	201	203	167	199	228	238
Paklenica	Pak34	203	225	157	199	238	238
Imotski	Im3	203	203	161	199	220	238
*Imotski*	*Im4*	*203*	*214*	*163*	*167*	*233*	***246***
Imotski	Im5	185	214	159	163	238	238
*Imotski*	*Im7*	*185*	*203*	*197*	*197*	***239***	***239***
Imotski	Im8	183	214	157	159	238	238
Imotski	Im11	203	214	159	167	228	238
*Imotski*	*Im14*	*185*	*214*	*159*	*197*	*238*	***239***
Imotski	Im17	203	214	159	161	220	238
Imotski	Im18	203	214	161	163	220	233
Imotski	Im19	203	214	163	199	233	238
Imotski	Im20	214	214	159	159	238	238
*Imotski*	*Im21*	*203*	*203*	*157*	*197*	*228*	***239***
Lukovdol	Luk1	201	214	159	159	238	238
*Lukovdol*	*Luk2*	*203*	*225*	*161*	*203*	*220*	***239***
Lukovdol	Luk3	201	203	159	167	238	244
Lukovdol	Luk4	201	203	167	197	238	238
*Lukovdol*	*Luk5*	*201*	*203*	*161*	*197*	*234*	***239***
Lukovdol	Luk6	201	203	167	167	238	238
*Lukovdol*	*Luk8*	*201*	*225*	*159*	*197*	***239***	***246***
*Lukovdol*	*Luk10*	*201*	*203*	*159*	*197*	*238*	***239***
*Lukovdol*	*Luk11*	*201*	*203*	*197*	*203*	*238*	***239***
Lukovdol	Luk12	203	203	159	203	238	238
Lukovdol	Luk13	201	203	197	203	233	238
Lukovdol	Luk14	201	214	197	199	234	238
*Lukovdol*	*Luk15*	*201*	*214*	*159*	*159*	*238*	***239***
Lukovdol	Luk16	201	203	199	203	233	238
Lukovdol	Luk17	201	203	167	167	233	233
*Lukovdol*	*Luk18*	*203*	*214*	*159*	*167*	*238*	***239***
Lukovdol	Luk19	201	201	159	197	233	238
*Lukovdol*	*Luk20*	*201*	*201*	*159*	*197*	*238*	***239***
*Grab*	*Grab1*	*185*	*203*	*163*	*197*	*233*	***239***
*Krka*	*Krka9*	*203*	*214*	*157*	*167*	*238*	***239***
Krka	Krka15	185	201	197	197	234	234
Krka	Krka18	185	203	161	197	220	234
Krka	Krka19	203	203	161	197	220	234
*Krka*	*Krka20*	*185*	*203*	*197*	*197*	*234*	***239***
Krka	Krka21	203	203	161	197	220	234
Krka	Krka24	185	185	161	167	220	238
Krka	Krka26	201	203	197	197	234	234
Krka	Krka27	185	203	161	197	220	234
Psunj	Psunj3	203	214	167	199	234	238
*Psunj*	*Psunj4*	*201*	*227*	*195*	*197*	*238*	***239***
Psunj	Psunj5	201	201	161	167	220	238
Psunj	Psunj7	203	214	159	161	220	238
*Psunj*	*Psunj8*	*201*	*203*	*167*	*197*	*238*	***239***
*Psunj*	*Psunj10*	*203*	*214*	*167*	*199*	*238*	***239***
*Psunj*	*Psunj11*	*203*	*214*	*197*	*197*	***239***	***239***
*Psunj*	*Psunj12*	*203*	*214*	*197*	*197*	***239***	***239***
*Psunj*	*Psunj14*	*201*	*214*	*167*	*199*	*238*	***239***
Psunj	Psunj21	201	203	163	167	233	238
*Psunj*	*Psunj22*	*201*	*214*	*197*	*199*	*238*	***239***
*Psunj*	*Psunj23*	*193*	*214*	*159*	*161*	*220*	***239***
*Psunj*	*Psunj24*	*201*	*201*	*201*	*203*	***239***	***239***
*Psunj*	*Psunj25*	*193*	*214*	*159*	*161*	*220*	***239***
*Psunj*	*Psunj26*	*193*	*214*	*159*	*161*	*220*	***239***
Psunj	Psunj27	193	201	161	167	220	238
*Psunj*	*Psunj28*	*214*	*214*	*159*	*167*	*238*	***239***
Gizdavac	Giz1	201	203	163	199	233	238
*Gizdavac*	*Giz2*	*185*	*203*	*163*	*197*	*233*	***239***

Rows in italics individuals carrying R-alleles. Allele 239 at marker SC47-18 (in bold) was linked to resistance in *V. sylvestris.* Allele 246 at marker SC47-18 (in bold) was previously linked to resistance in *V. vinifera*^7^.

None of the in situ-tested individuals had R-alleles at SC8-0071-014 and UDV124, despite polymorphisms at both markers (Table 2).

**Figure 3:**
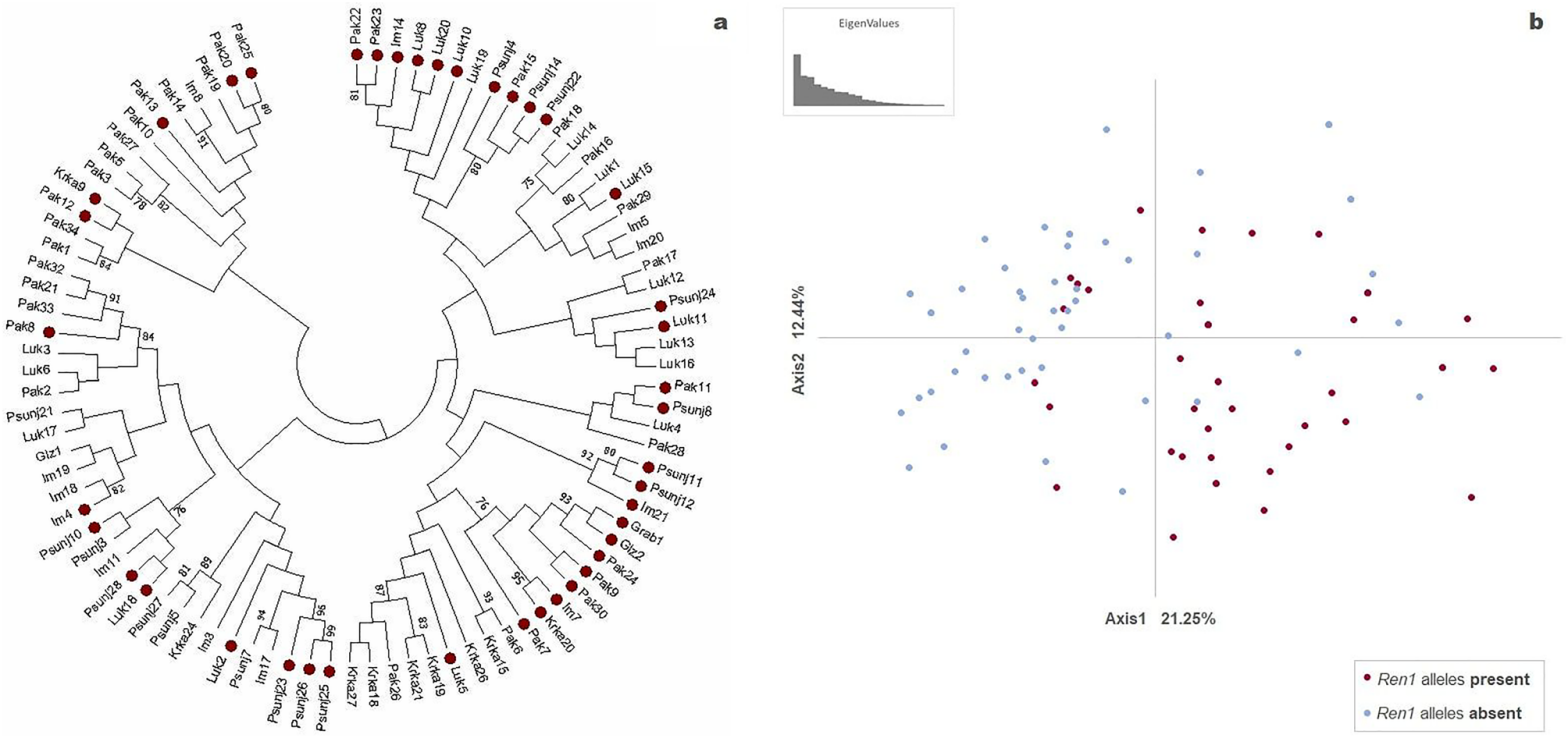
Clustering of 91 wild grapevine individuals based on three SSR markers at the *Ren1* locus. (**a)** Neighbor-joining dendrogram showing genetic relationships among individuals with bootstrap support value ≥ 75%. The samples with resistance alleles are marked with red dots. Analyses were conducted in MEGA7^21^. (**b)** Principal coordinate (PCoA) projections of individuals in a 2-D plot based on genotypic distances conducted in GenAlex^22^.

The set of 91 in situ-tested individuals was subjected to neighbor-joining (NJ) clustering and perceptual mapping (Principal Coordinate Analysis, PCoA) to visualize the similarity among individuals based on SSR allelic profiles (Fig. 3a,b). The NJ tree showed clusters of mixed individuals from different populations, only roughly outlining their geographical origin. However, individuals with R-alleles are clearly more abundant on the right side of the tree (red dots, Fig. 3a). Relative relationships between individuals based on PCoA showed the same pattern as NJ. They separated individuals with R- alleles on the right side of plot from individuals without R-alleles at *Ren1* on the left side. PCoA projections of the first two dimensions accounted for 33.69% of the total molecular variation. PCoA revealed slight overlapping between the two groups (Fig. 3b).

### Genetic diversity

Genetic diversity was calculated for the two sets of *V. sylvestris*: 91 in situ individuals and 67 seedlings. Three SSR markers were polymorphic in both sets (Table 3). The observed heterozygosity was lower than the expected heterozygosity, except at marker UDV124 and for the in situ set at marker SC47-18. The in situ set showed more heterozygosity at the tested markers than the seedling set. The allele frequency (AF) for resistance-related allele 212 at UDV124 was determined only in the seedling set with (AF = 0.04). At marker SC8-0071-014, no resistance-linked alleles were observed. The AF was lower than 0.3 for all alleles except for the 197 allele in seedlings, which was present in the entire Cerovica set (Table 1). The greatest abundance of R-alleles was found at marker SC47-18: R-allele 239 was found in the in situ and seedling sets at an AF of 0.25 and 0.26, respectively. An additional R-allele 246 was observed in the in situ set only, at a very low AF of 0.01.

**Table 3:** Genetic diversity at three SSR markers linked to the *Ren1* locus for powdery mildew resistance in *V. sylvestris.*

Locus		Na	Ho	He	Allele	Allele frequency	
						In situ *V. sylvestris*	*V. sylvestris* seedlings
**UDV124**	In situ *V. sylvestris*	8	0.84	0.79	183	0.03	
	*V. sylvestris* seedlings	8	0.82	0.72	185	0.14	0.24
					193	0.02	
					201	0.28	0.12
					203	0.30	0.45
					210		0.05
					**212**		**0.04**
					214	0.15	0.08
					216		0.01
					225	0.07	0.02
					227	0.01	
**SC8-0071-014**	In situ *V. sylvestris*	11	0.82	0.84	157	0.08	0.05
	*V. sylvestris* seedlings	8	0.69	0.77	159	0.15	0.13
					161	0.09	
					163	0.06	0.15
					166	0.01	
					167	0.19	0.08
					171		0.01
					195	0.01	
					197	0.26	0.38
					199	0.10	0.20
					201	0.01	
					203	0.04	0.01
**SC47-18**	In situ *V. sylvestris*	8	0.75	0.75	213		0.03
	*V*. *sylvestris* seedlings				220	0.09	
		6	0.72	0.73	228	0.03	
					233	0.09	0.24
					234	0.11	0.09
					237		0.01
					238	0.41	0.37
					**239**	**0.25**	**0.26**
					244	0.02	
					**246**	**0.01**	

The alleles in bold are linked to resistance at *Ren1* according to Riaz et al.^7,15^.

'Na' is the number of different alleles, 'Ho' is the observed heterozygosity, and 'He' is the expected heterozygosity.

## Discussion

**Figure 4:**
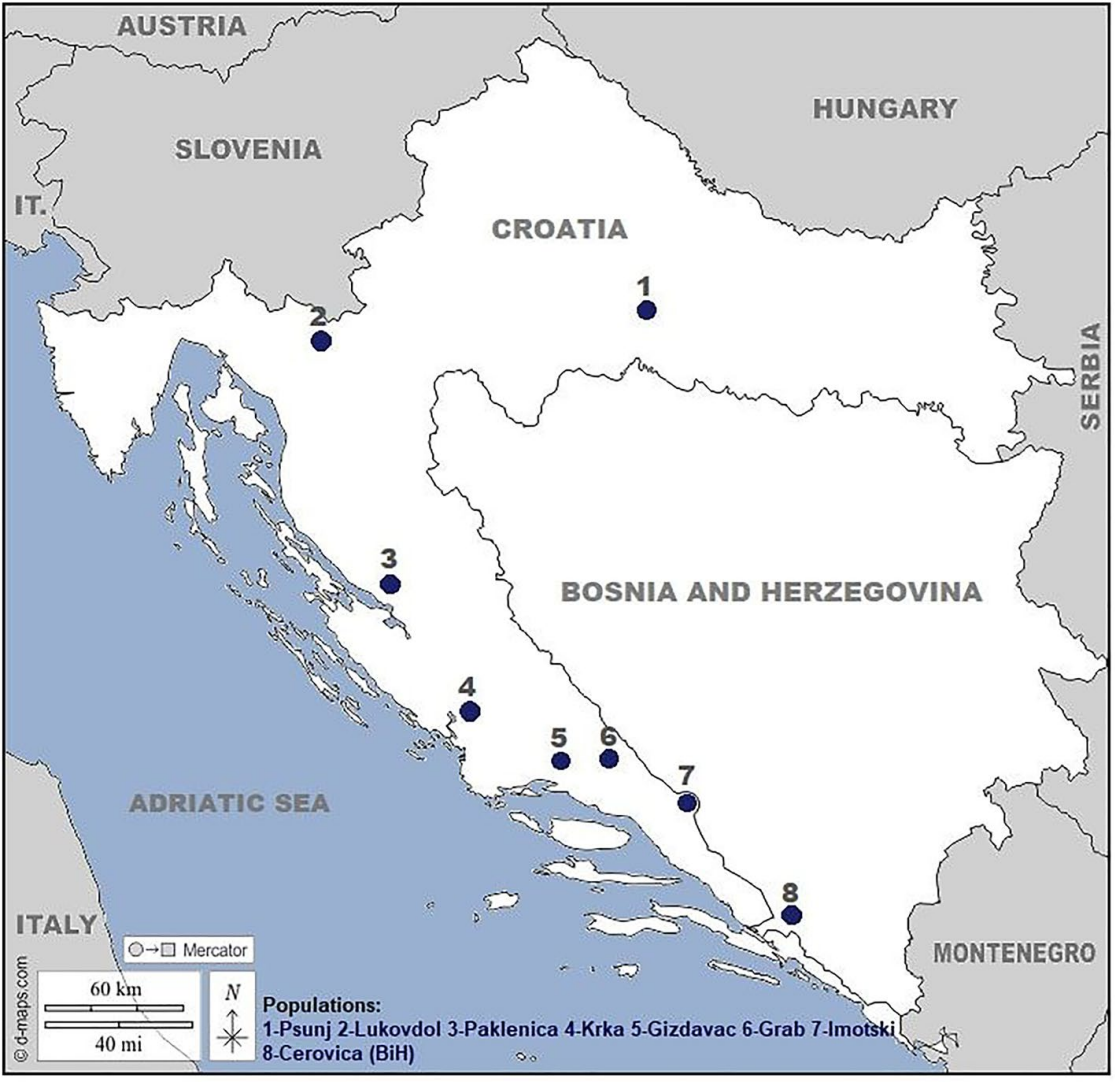
Map of seven in situ Croatian *V. sylvestris* populations and the Cerovica population in Bosnia and Herzegovina.

This study represents the first screening for powdery mildew resistance in eastern Adriatic *V. sylvestris* germplasm. The phenotypic focus of study was on the *V. sylvestris* seedlings in an ex situ collection. These were young potted plants evaluated for powdery mildew resistance under in vivo and in vitro conditions*.* The resistance level of seedlings varied from very susceptible to medium to highly resistant. The control cultivars in this study showed medium to very high resistance (OIV455 scores from 5 to 9), except cvs. ‘Rukatac’ and ‘Pošip bijeli’ in 2019. These white grape cultivars are considered susceptible to fungal diseases. None of the control cultivars had R-alleles at the three SSR markers, even though cv. ‘Plavac mali sivi’ is known to have good tolerance toward fungal infections. Control cultivars clearly expressed more robust vigour than *V. sylvestris* accessions. Twenty-two and 23 in vivo-evaluated seedlings showed high resistance to PM in 2018 and 2019, respectively. The majority of seedlings showed PM infection, indicating homogeneous field infections. Seedlings from the Cerovica population (Bosnia and Herzegovina; Fig. 4, Table 1) had the greatest average resistance and the most accessions with R-alleles (23 out of 27 accessions). Cerovica, which is part of the large Neretva population in Bosnia and Herzegovina, contained a considerable number of different and private alleles^20^ and possibly retained an important fraction of the historic *V. sylvestris* biodiversity. The seedlings from Paklenica and Gizdavac had intermediate resistance. The Paklenica population is one of the most-conserved *V. sylvestris* populations in Croatia and had the most R-alleles in its in situ accessions, while Gizdavac is one of the most vulnerable populations, close to urban centers and with visible human impact^19,23^. Seedlings from Gizdavac had greater allelic diversity of SSR markers than those from Paklenica (the least), even though the overall phenotypic resistance of Gizdavac seedlings was the lowest in this study.

During in vivo evaluation, nine seedlings were confirmed as resistant in both years. Of these nine, three were susceptible in the leaf disk assay. Seedling SjCer7 had the greatest leaf disk resistance and carried R-allele 212 at marker UDV124. This allele is associated with resistance in *V. vinifera *^6^. On the other hand, four seedlings were susceptible both years in vivo. One had a single R-allele 212, two had a single R-allele 239 and one had no R-alleles.

PM resistance (score 7) in the seedling subset leaf disk assay was confirmed in both years for only three accessions from the Cerovica population. The resistant accessions SjCer14, carrying the single allele 239, and SjCer23, carrying two alleles (212 and 239), had the same phenotypic disease scores regardless of the evaluation method and were slightly more resistant than SjCer24, the resistant accession without R-alleles. Among the entire seedling set, only two accessions from Cerovica, SjCer1 and SjCer23, had R-alleles at two markers (UDV124 and SC47-18) and expressed PM resistance.

In general, disease evaluations are very subjective and vary among studies due to different environmental conditions, experimental set up, disease pressure, and pathogen population structure. The phenotypic evaluations conducted in this study differed to some extent from Riaz et al.^7^, in which a modified and inverted OIV scale was used. Similar modifications were reported in other studies^8,10,24,25^. The seedling plants in this study were very young at the time of phenotypic evaluations, which probably affected both the level of resistance and disease pressure to some extent. Young plants do not have excess vegetative growth, which allows more air circulation and potentially reduces infection severity. Nevertheless, the final outcomes were comparable. In this study, most seedlings showed intermediate resistance to PM, characterized by clearly visible, but localized and fragmented, sporulation with small patches of mycelium. However, in the 2018 in vitro analysis, most seedlings expressed resistance to PM. Mycelial growth was highly suppressed and conidiospores were not observed. The resistance of the seedling set under in vitro conditions varied greatly between the two seasons (14 and 57% of the accessions expressed resistance). Under in vivo conditions, unsprayed, naturally-infected plants showed more consistency between seasons, with the number of resistant seedlings varying between 35 and 40%. This variation might be because of using more mature leaves in the assay and variation in the PM inoculum, which was collected from the field. Field PM strains can vary from one year to another^26^, which might cause variations in resistance levels. In our samples, intermediate resistance was predominant. This differed from phenotyping results of Riaz et al.^7^, where the intermediate rating was not observed among the seedlings. This was probably due to more consistent in vitro assay infections and inoculation with a single PM strain collected from pure cultures^7^. In our study, randomly-collected, spontaneous field PM inoculum was used. Moreover, full parentage was known for only nine seedlings (23% of the seedling set), thus information on the phenotypic resistance to PM of the in situ parental lineages is entirely missing. In a previous study^7^, the F1 population of seedlings resulted from crosses between known resistant (*V. sylvestris*) and susceptible (*V. vinifera*) parents. Research on ‘omics’ provides additional and unique insight into the linking of phenotypes with genotype interactions and plant stress responses^27^. Comprehensive research on the transcriptomics of Central Asian accessions revealed varying levels of phenotypic resistance to PM, matched with various transcriptomic responses to *E. necator* among Central Asian accessions^24^.

The three *Ren1*-linked SSR markers on chromosome 13 that we analyzed in situ in Croatian *V. sylvestris* populations and in seedlings were highly polymorphic. The observed heterozygosity was lower than the expected heterozygosity, indicating lower genetic diversity among the studied sets at the three SSR markers. This is similar to what Riaz et al.^7^ found at four (of five) R-SSR loci for the Central Asian grape germplasm, pointing to inbreeding due to geographic isolation of the populations. Increased gene flow might be assumed, for instance, in seedlings from Gizdavac, where out of two individuals, only the male parent, Giz2, had the R-allele 239, which was not found in its progeny (SjGiz4). Eight seedlings from Gizdavac had various R-alleles that were most likely inherited from their unknown paternal parents. The Gizdavac population, near urban centers, differs from the protected Paklenica population, where most in situ individuals had an R allele. Only two progeny seedlings had an R-allele, 239, which they shared with their mother accession, Pak12. SNP Genotyping by Sequencing approach revealed significant and unexpectedly high segregation distortions from Mendelian ratios on chromosome 13 in the susceptible *V. vinifera* cv. ‘Glera’, making one end of its chromatide less-inherited in the offspring^28^.

In this study, none of the accessions had R-alleles (141 or 143) at the SC8-0071-14 locus. This locus was previously shown to be polymorphic and a significant Quantitative trait loci (QTL), explaining up to 96% of the variation. It was mapped together with the SC47-18 locus. In a previous study^7^, SC8-0071-014 R-alleles were detected in both resistant and susceptible accessions. Here, accessions carrying R-alleles at UDV124 and SC47-18 were either resistant or susceptible. Locus UDV124 flanks *Ren1* on chromosome 13, while loci SC8-0071–014 and SC47-18 co-segregated with resistance (*Ren1*)^29^. Clustering by NJ and PCoA both separated individuals carrying R-alleles to the right side of the graphs (Fig. 3a,b). There was no clear phylogenetic separation of individuals by population of origin, although there was rough separation between northern and southern populations (Fig. 3a). PCoA more clearly visualized the overlapp between two groups. These results may indicate a common source of *Ren1* loci in studied *V. sylvestris*. However, as was recently discussed for Caucasus *V. vinifera* cvs., high complexity at the chromosome 13 region and information gathered thus far does not permit conclusions as to whether studied genotypes from the Caucasus and Central Asia share the same resistance genes^28^.

This study provides insight into powdery mildew resistance of *V. sylvestris* accessions from the eastern Adriatic region. The observed phenotypic resistance of *V. sylvestris* individuals to powdery mildew was clear and consistent for some individuals, but showed some weakness in connecting phenotypic and genetic resistance in other individuals. The SC47-18 marker (R-allele 239) co-segregated with resistance at *Ren1.* It was the most dominant marker in our *V. sylvestris* set and there were no R-alleles at another co-segregating marker, SC8-0071-014. However, a trend with two new allelic combinations was found in the studied set. This has interest for a deeper investigation on functional PM resistance related to that marker and the *Ren1* locus. No substantial connection was observed between individuals with greater phenotypic resistance and expected resistant genotypes. This result was in accordance with previous results, in which both resistant and susceptible genotypes had R-alleles. However, accessions with two R-alleles at two different SSR markers showed, on average, greater resistance to powdery mildew in this study. The presence of R-alleles in the eastern Adriatic *V. sylvestris* confirms these genetic resources as important sources of biodiversity. Next-generation sequencing technologies and other –omics methods would be beneficial to access more information on the nature of PM resistance in the eastern Adriatic region.

## Methods

### Plant material

A total of 158 unique *V. sylvestris* genotypes were analyzed in this study. The sample set consisted of 91 V*. sylvestris* genotypes from seven natural populations in Croatia (in situ) and 67 V*. sylvestris* seedlings established from seeds of five *V. sylvestris* plants from three natural populations: Paklenica and Gizdavac in Croatia and Cerovica in Bosnia-Herzegovina. All *V. sylvestris* individuals included in this study were identified morphologically and using 20 SSR markers through our previous inventorying of *V. sylvestris* in Croatia and neighboring countries^18,20,30^. Permission to collect and examine the plant species *Vitis vinifera* subsp. *sylvestris* within protected natural areas of Croatia has been granted by the Ministry of Economy and Sustainable Development of the Republic of Croatia (Permission No. UP/I-612-07/15-33/74). The voucher specimens were identified by Goran Zdunić and Katarina Lukšić and are deposited in the publicly accessible herbarium of the Institute for Adriatic Crops and Karst Reclamation, Split, Croatia.

The natural habitats of the studied species cover diverse geographic areas: the eastern coast of the Adriatic Sea, the mountainous area of the Dinaric Alps, and the mountainous area in the Croatian part of the Pannonian Basin. Supplementary Table S1 lists the analyzed genotypes, their geographical origins and their sampling locations. The seven natural *V. sylvestris* populations were from Imotski, Grab, Gizdavac, Krka, Paklenica, Lukovdol and Psunj (Fig. 4).

### Disease evaluations

PM symptoms were first observed on wild individuals in situ during an inventory of *V. sylvestris* around bloom. The overall health status of each plant was evaluated. PM symptoms on green tissues, including leaves (adaxial surfaces), were noted: white to grayish coatings of fungal colonies, upward leaf curling, and leaves that were drying and falling off.

The in situ* V. sylvestris* plants were not subjected to leaf disk assays in this study due to their spatial dislocation and the difficulty of keeping the plant material fresh and ensuring uniform trial conditions.

PM evaluation of the 67 V*. sylvestris* seedlings was performed (with no biological replicates) on plants and via a leaf disk assay (only 35 seedlings) using the OIV 455 descriptor^31^. Each genotype was evaluated by visual inspection for signs of pathogens using a stereomicroscope and the OIV 455 scale: 1 = unlimited infection; complete or nearly complete attack of the leave surface, abundant mycelial growth 3 = vast attacked patches, some of which were limited, obvious mycelial growth and fungus fructification; 5 = attacked patches were frequent, but usually clearly limited; 7 = sparse, small and limited attacked patches; little mycelium and fungus fructification, 9 = greatly suppressed symptoms or none at all; no mycelium or visible fructification.

The seedlings were grown in pots in an outdoor shaded area and were not sprayed from the beginning of vegetation, before and during the PM evaluations. The disease evaluations were conducted during 2018 and 2019.

The subset of 35 out of 67 seedlings selected for the in vitro leaf disk assays included only individuals with the best health status and overall fitness.

The in vivo PM evaluations were performed on May 21^st^ in 2018 and on May 13^th^ in 2019, during the highest disease pressure in the field collection period. The observations encompassed 67 V*. sylvestris* seedlings and the cv. 'Plavac mali sivi' as a control with three accessions. The in vivo inspections were carried out by evaluating an approximately equal part of each plant and taking into consideration the overall plant health, after which a single OIV 455 score was assigned per genotype.

For the leaf disk analyses, besides 35 V*. sylvestris* samples (Table 1), four potted cvs. 'Plavac mali sivi' (1 replicate), 'Pošip bijeli' (2 replicates) and 'Rukatac' (1 replicate), were included as references. The pink cv. 'Plavac mali sivi' is generally known to be less susceptible to mildew, while the white cvs. 'Rukatac' and 'Pošip bijeli' are susceptible.

### Leaf disk assays

The in vitro analyses were performed by placing leaf disks on 1% water-agar (Bacto™ Agar, BD, France) in plastic Petri dishes, as described^8,32^. The leaf disks were infected with *E. necator* using fresh conidia from naturally infected leaves of susceptible *V. sylvestris* seedlings grown in the collection. The spores were collected with a small soft brush and transferred onto each adaxial leaf surface. The fourth to sixth fully developed leaves of each genotype were sampled: two leaves (four leaf disks) were analyzed per sample. The samples were placed in a climate chamber at 25 °C and 65% relative humidity on a 16-h light, 8-h dark cycle. Seven to nine *days post inoculation* (dpi), leaves were evaluated for mycelial growth and conidiophore formation. Evaluations of the infections were performed using a CarlZeiss stereomicroscope (Microimaging, GmbH Germany), and detailed inspections together with photos were captured under 0.65 × magnification.

### DNA extraction and marker analysis

DNA was extracted from the young leaves using the NucleoSpin Plant II kit (Macherey–Nagel, Düren, Germany). The extracted DNA was quantified and used at a working DNA concentration of 1 ng/μL.

Three microsatellite markers, SC47-18, SC8–0071–14 and UDV124^6^, that are associated with the *Ren1* gene for PM resistance were analyzed. The SSR markers were multiplexed within one run. All forward primers were labeled on the 5' end with fluorescent dyes (NED, 6-FAM and VIC).

A KAPA Fast Multiplex PCR Kit (2x) (Kapa Biosystems, Wilmington, MA) was used to set up 5-µL volume reaction mixtures containing a master mix, 100 pmol of each primer, and ~ 1 ng template DNA. PCR amplification was performed using the following program: three minutes of initial denaturation at 95 °C, followed by 30 cycles of denaturation at 95 °C (15 s), annealing at 60 °C (30 s), and extension at 72 °C (30 s). A final extension was performed at 72 °C for seven min.

The amplified products were resolved using capillary electrophoresis on an ABI 3730xl Genetic Analyzer (Applied Biosystems) using GeneScan-LIZ 500 as an internal standard. The peaks were identified with GeneMapper 4.0 software (Applied Biosystems). The allele calling was harmonized with previously published SSR data^7,15^ by amplifying two identical *V. sylvestris* samples separately at the University of California at Davis and at the Laboratory of the IAC in Split. Then, the allele sizes were aligned among the other samples.

The *trueness to type* of the *V. sylvestris* set was analyzed and discussed in a previous study^30^.

### Data analysis

The phenotypic data and corresponding statistical analyses were performed in Excel 2013. The disease scores were determined according to the OIV 455 scale: by calculating mode values from four leaf disk scores per genotype each year and by determining a direct, single OIV score per genotype for all in vivo evaluations each year.

Descriptive statistics of the SSR markers used in this study were calculated for the following indices: the number of different alleles per locus (Na), expected heterozygosity (He), observed heterozygosity (Ho) and allele frequency (AF). These statistics were obtained using the GenAlEx 6.5 package^22^. Principal coordinate analysis (PCoA) was performed to visualize relationships between individuals based on the *Ren1* scoring data via covariance, standardized using the same program.

The SSR data for 91 in situ individuals were analyzed to compute their evolutionary history using the Neighbor-Joining method^33^ implemented in MEGA 7.0 software^21^. The bootstrap interior branch test was used to test the reliability of each interior branch on the tree^34^.

## Supplementary Information

Supplementary Table S1.

## Data Availability

All data generated or analyzed during this study are included in this published article (and its Supplementary Information files). Correspondence and requests for materials should be addressed to G.Z. The study complies with local and national regulations.
