# Supplementary material for: Identification of powdery mildew resistance in wild grapevine (Vitis vinifera subsp. sylvestris Gmel Hegi) from Croatia and Bosnia and Herzegovina
Source: Sci Rep. 2022 Feb 8;12:2128. doi: 10.1038/s41598-022-06037-6 (PMC8826913; doi:10.1038/s41598-022-06037-6)
Supplement: Supplementary file 1 — Supplementary Table S1. [file 41598_2022_6037_MOESM1_ESM.pdf]

Supplement information

Manuscript title:

Identification of powdery mildew resistance in wild grapevine (*Vitis vinifera* subsp. *sylvestris* Gmel Hegi) from Croatia and Bosnia and Herzegovina

Authors: Katarina Lukšić[1], Goran Zdunić[1]*, Katarina Hančević[1], Maja Žulj Mihaljević[2], Ana Mucalo[1], Erika Maul[3], Summaira Riaz[4], Ivan Pejić[2,5]

Institutions:

[1] Institute for Adriatic Crops and Karst Reclamation, Put Duilova 11, 21 000 Split, Croatia

[2] University of Zagreb, Faculty of Agriculture, Svetošimunska cesta 25, 10 000 Zagreb, Croatia

[3] Julius Kühn-Institute, Federal Research Centre for Cultivated Plants, Institute for Grapevine Breeding Geilweilerhof, 76833 Siebeldingen, Germany

[4] Department of Viticulture and Enology, University of California, Davis, CA 95616, USA

[5] Centre of Excellence for Biodiversity and Molecular Plant Breeding, 10 000 Zagreb, Croatia

Supplementary Table S1.

List of 158 *V. sylvestris* individuals analyzed in this study. Set of 91 *in situ* individuals from natural habitats and 67 seedlings from an ex situ collection (IAC) in Split (Croatia) were genotyped at three SSR markers: SC47-18, SC8-071-0014, and UDV-124 linked to *Ren1* locus on chromosome 13 for powdery mildew resistance. Set of *V. sylvestris* seedlings was additionally subjected to phenotypic powdery mildew resistance analysis according to descriptor OIV 455 using *in vivo* and leaf disk evaluation methods.

Population	Species	GPS	Genotype
**91 *V. sylvestris in situ* genotypes**			
Paklenica	*Vitis v.subsp. sylvestris*	N44º17' 914" E15º 27' 831"	Pak1
Paklenica	*Vitis v.subsp. sylvestris*	N44º17' 910" E15º 27' 802"	Pak2
Paklenica	*Vitis v.subsp. sylvestris*	N44º17' 934" E15º 28' 121"	Pak3
Paklenica	*Vitis v.subsp. sylvestris*	N44º17' 915" E15º 28' 165"	Pak5
Paklenica	*Vitis v.subsp. sylvestris*	N44º17' 994" E15º28' 188"	Pak6
Paklenica	*Vitis v.subsp. sylvestris*	N44º17' 977" E15º28' 177"	Pak7
Paklenica	*Vitis v.subsp. sylvestris*	N44º17' 984" E15º28' 171"	Pak8
Paklenica	*Vitis v.subsp. sylvestris*	N44º18' 013" E15º28' 188"	Pak9
Paklenica	*Vitis v.subsp. sylvestris*	N44º18' 028" E15º28' 209"	Pak10
Paklenica	*Vitis v.subsp. sylvestris*	N44º18' 042" E15º28' 218"	Pak11
Paklenica	*Vitis v.subsp. sylvestris*	N44º18' 260" E15º28' 318"	Pak12
Paklenica	*Vitis v.subsp. sylvestris*	N44º18' 348" E15º28' 452"	Pak13
Paklenica	*Vitis v.subsp. sylvestris*	N44º18' 402" E15º28' 480"	Pak14
Paklenica	*Vitis v.subsp. sylvestris*	N44º18' 400" E15º28' 467"	Pak15
Paklenica	*Vitis v.subsp. sylvestris*	N44º18' 476" E15º28' 384"	Pak16
Paklenica	*Vitis v.subsp. sylvestris*	N44º18' 670" E15º28' 298"	Pak17
Paklenica	*Vitis v.subsp. sylvestris*	N44º18' 463" E15º28' 591"	Pak18
Paklenica	*Vitis v.subsp. sylvestris*	N44º18' 351" E15º28' 635"	Pak19
Paklenica	*Vitis v.subsp. sylvestris*	N44º18' 465" E15º28' 706"	Pak20
Paklenica	*Vitis v.subsp. sylvestris*	N44º18' 465" E15º28' 705"	Pak21
Paklenica	*Vitis v.subsp. sylvestris*	N44º18' 493" E15º28' 743"	Pak22
Paklenica	*Vitis v.subsp. sylvestris*	N44º18' 508" E15º28' 749"	Pak23
Paklenica	*Vitis v.subsp. sylvestris*	N44º18' 642" E15º28' 811"	Pak24
Paklenica	*Vitis v.subsp. sylvestris*	N44º18' 689" E15º28' 795"	Pak25
Paklenica	*Vitis v.subsp. sylvestris*	N44º18' 700" E15º28' 799"	Pak26
Paklenica	*Vitis v.subsp. sylvestris*	N44º18' 774" E15º28' 802"	Pak27
Paklenica	*Vitis v.subsp. sylvestris*	N44º19' 242" E15º28' 675"	Pak28
Paklenica	*Vitis v.subsp. sylvestris*	N44º19' 254" E15º28' 671"	Pak29
Paklenica	*Vitis v.subsp. sylvestris*	N44º19' 526" E15º28' 655"	Pak30
Paklenica	*Vitis v.subsp. sylvestris*	N44º17' 919" E15º27' 868"	Pak32
Paklenica	*Vitis v.subsp. sylvestris*	N44º18' 375" E15º28' 417"	Pak33
Paklenica	*Vitis v.subsp. sylvestris*	N44º18' 721" E15º28' 351"	Pak34
Imotski	*Vitis v.subsp. sylvestris*	N43º26' 958" E17º12' 541"	Im3
Imotski	*Vitis v.subsp. sylvestris*	N43º26' 956" E17º12' 534"	Im4
Imotski	*Vitis v.subsp. sylvestris*	N43º26' 955" E17º12' 539"	Im5
Imotski	*Vitis v.subsp. sylvestris*	N43º26' 965" E17º12' 513"	Im7
Imotski	*Vitis v.subsp. sylvestris*	N43º26' 955" E17º12' 584"	Im8
Imotski	*Vitis v.subsp. sylvestris*	N43º26' 969" E17º12' 524"	Im11
Imotski	*Vitis v.subsp. sylvestris*	N43º27' 041" E17º12' 574"	Im14
Imotski	*Vitis v.subsp. sylvestris*	N43º26' 996" E17º12' 515"	Im17

Imotski	*Vitis v.subsp. sylvestris*	N43º26' 994" E17º12' 514"	Im18
Imotski	*Vitis v.subsp. sylvestris*	N43º26' 997" E17º12' 526"	Im19
Imotski	*Vitis v.subsp. sylvestris*	N43º27' 093" E17º12' 619"	Im20
Imotski	*Vitis v.subsp. sylvestris*	N43º27' 085" E17º12' 621"	Im21
Lukovdol	*Vitis v.subsp. sylvestris*	nd	Luk1
Lukovdol	*Vitis v.subsp. sylvestris*	nd	Luk2
Lukovdol	*Vitis v.subsp. sylvestris*	N45º26' 160" E15º08' 035"	Luk3
Lukovdol	*Vitis v.subsp. sylvestris*	N45º26' 143" E15º08' 045"	Luk4
Lukovdol	*Vitis v.subsp. sylvestris*	N45º26' 140" E15º08' 044"	Luk5
Lukovdol	*Vitis v.subsp. sylvestris*	N45º26' 131" E15º08' 023"	Luk6
Lukovdol	*Vitis v.subsp. sylvestris*	N45º26' 169" E15º08' 048"	Luk8
Lukovdol	*Vitis v.subsp. sylvestris*	N45º26' 182" E15º08' 181"	Luk10
Lukovdol	*Vitis v.subsp. sylvestris*	N45º26' 120" E15º08' 194"	Luk11
Lukovdol	*Vitis v.subsp. sylvestris*	N45º26' 110" E15º08' 209"	Luk12
Lukovdol	*Vitis v.subsp. sylvestris*	N45º26' 098" E15º08' 276"	Luk13
Lukovdol	*Vitis v.subsp. sylvestris*	N45º26' 085" E15º08' 277"	Luk14
Lukovdol	*Vitis v.subsp. sylvestris*	N45º26' 232" E15º08' 373"	Luk15
Lukovdol	*Vitis v.subsp. sylvestris*	nd	Luk16
Lukovdol	*Vitis v.subsp. sylvestris*	N45º26' 246" E15º08' 232"	Luk17
Lukovdol	*Vitis v.subsp. sylvestris*	N45º26' 250" E15º08' 185"	Luk18
Lukovdol	*Vitis v.subsp. sylvestris*	N45º26' 269" E15º08' 154"	Luk19
Lukovdol	*Vitis v.subsp. sylvestris*	N45º26' 262" E15º08' 150"	Luk20
Grab	*Vitis v.subsp. sylvestris*	N43º38' 454" E16º46' 223"	Grab1
Krka	*Vitis v.subsp. sylvestris*	N43º48' 069" E15º58' 210"	Krka9
Krka	*Vitis v.subsp. sylvestris*	N43º48' 341" E15º58' 013"	Krka15
Krka	*Vitis v.subsp. sylvestris*	N43º48' 295" E15º58' 162"	Krka18
Krka	*Vitis v.subsp. sylvestris*	N43º48' 298" E15º58' 206"	Krka19
Krka	*Vitis v.subsp. sylvestris*	nd	Krka20
Krka	*Vitis v.subsp. sylvestris*	N43º48' 357" E15º58' 392"	Krka21
Krka	*Vitis v.subsp. sylvestris*	N43º48' 253" E15º 57' 949"	Krka24
Krka	*Vitis v.subsp. sylvestris*	N43º48' 187" E15º 57' 995"	Krka26
Krka	*Vitis v.subsp. sylvestris*	N43º48' 276" E15º58' 008"	Krka27
Psunj	*Vitis v.subsp. sylvestris*	nd	Psunj3
Psunj	*Vitis v.subsp. sylvestris*	nd	Psunj4
Psunj	*Vitis v.subsp. sylvestris*	nd	Psunj5
Psunj	*Vitis v.subsp. sylvestris*	N45º23' 405" E17º27' 524"	Psunj7
Psunj	*Vitis v.subsp. sylvestris*	N45º23' 383" E17º27' 593"	Psunj8
Psunj	*Vitis v.subsp. sylvestris*	N45º23' 375" E17º27' 639"	Psunj10
Psunj	*Vitis v.subsp. sylvestris*	N45º23' 450" E17º27' 313"	Psunj11
Psunj	*Vitis v.subsp. sylvestris*	N45º23' 448" E17º27' 322"	Psunj12
Psunj	*Vitis v.subsp. sylvestris*	N45º23' 445" E17º27' 321"	Psunj14
Psunj	*Vitis v.subsp. sylvestris*	N45º23' 446" E17º27' 347"	Psunj21
Psunj	*Vitis v.subsp. sylvestris*	N45º23' 429" E17º27' 354"	Psunj22
Psunj	*Vitis v.subsp. sylvestris*	N45º23' 499" E17º28' 071"	Psunj23
Psunj	*Vitis v.subsp. sylvestris*	N45º23' 501" E17º28' 059"	Psunj24
Psunj	*Vitis v.subsp. sylvestris*	N45º23' 503" E17º28' 056"	Psunj25
Psunj	*Vitis v.subsp. sylvestris*	N45º23' 498" E17º28' 023"	Psunj26
Psunj	*Vitis v.subsp. sylvestris*	N45º23' 500" E17º28' 043"	Psunj27
Psunj	*Vitis v.subsp. sylvestris*	N45º23' 649" E17º28' 320"	Psunj28
Gizdavac	*Vitis v.subsp. sylvestris*	N43º39' 334" E16º29' 302"	Giz1

Gizdavac	*Vitis v.subsp. sylvestris*	nd	Giz2
**67 *V. sylvestris* seedlings**			
Gizdavac	*Vitis v.subsp. sylvestris*	IAC collection	SjGiz1
Gizdavac	*Vitis v.subsp. sylvestris*	IAC collection	SjGiz2
Gizdavac	*Vitis v.subsp. sylvestris*	IAC collection	SjGiz3
Gizdavac	*Vitis v.subsp. sylvestris*	IAC collection	SjGiz4
Gizdavac	*Vitis v.subsp. sylvestris*	IAC collection	SjGiz5
Gizdavac	*Vitis v.subsp. sylvestris*	IAC collection	SjGiz6
Gizdavac	*Vitis v.subsp. sylvestris*	IAC collection	SjGiz7
Gizdavac	*Vitis v.subsp. sylvestris*	IAC collection	SjGiz8
Gizdavac	*Vitis v.subsp. sylvestris*	IAC collection	SjGiz9
Gizdavac	*Vitis v.subsp. sylvestris*	IAC collection	SjGiz10
Gizdavac	*Vitis v.subsp. sylvestris*	IAC collection	SjGiz11
Gizdavac	*Vitis v.subsp. sylvestris*	IAC collection	SjGiz12
Gizdavac	*Vitis v.subsp. sylvestris*	IAC collection	SjGiz13
Gizdavac	*Vitis v.subsp. sylvestris*	IAC collection	SjGiz14
Gizdavac	*Vitis v.subsp. sylvestris*	IAC collection	SjGiz15
Gizdavac	*Vitis v.subsp. sylvestris*	IAC collection	SjGiz16
Gizdavac	*Vitis v.subsp. sylvestris*	IAC collection	SjGiz17
Gizdavac	*Vitis v.subsp. sylvestris*	IAC collection	SjGiz18
Gizdavac	*Vitis v.subsp. sylvestris*	IAC collection	SjGiz19
Gizdavac	*Vitis v.subsp. sylvestris*	IAC collection	SjGiz20
Gizdavac	*Vitis v.subsp. sylvestris*	IAC collection	SjGiz21
Gizdavac	*Vitis v.subsp. sylvestris*	IAC collection	SjGiz22
Gizdavac	*Vitis v.subsp. sylvestris*	IAC collection	SjGiz23
Gizdavac	*Vitis v.subsp. sylvestris*	IAC collection	SjGiz24
Gizdavac	*Vitis v.subsp. sylvestris*	IAC collection	SjGiz25
Paklenica	*Vitis v.subsp. sylvestris*	IAC collection	SjPak1
Paklenica	*Vitis v.subsp. sylvestris*	IAC collection	SjPak2
Paklenica	*Vitis v.subsp. sylvestris*	IAC collection	SjPak3
Paklenica	*Vitis v.subsp. sylvestris*	IAC collection	SjPak4
Paklenica	*Vitis v.subsp. sylvestris*	IAC collection	SjPak5
Paklenica	*Vitis v.subsp. sylvestris*	IAC collection	SjPak9
Paklenica	*Vitis v.subsp. sylvestris*	IAC collection	SjPak10
Paklenica	*Vitis v.subsp. sylvestris*	IAC collection	SjPak11
Paklenica	*Vitis v.subsp. sylvestris*	IAC collection	SjPak12
Paklenica	*Vitis v.subsp. sylvestris*	IAC collection	SjPak13
Paklenica	*Vitis v.subsp. sylvestris*	IAC collection	SjPak14
Paklenica	*Vitis v.subsp. sylvestris*	IAC collection	SjPak15
Paklenica	*Vitis v.subsp. sylvestris*	IAC collection	SjPak16
Paklenica	*Vitis v.subsp. sylvestris*	IAC collection	SjPak17
Paklenica	*Vitis v.subsp. sylvestris*	IAC collection	SjPak18
Cerovica	*Vitis v.subsp. sylvestris*	IAC collection	SjCer1
Cerovica	*Vitis v.subsp. sylvestris*	IAC collection	SjCer2
Cerovica	*Vitis v.subsp. sylvestris*	IAC collection	SjCer3
Cerovica	*Vitis v.subsp. sylvestris*	IAC collection	SjCer4
Cerovica	*Vitis v.subsp. sylvestris*	IAC collection	SjCer5
Cerovica	*Vitis v.subsp. sylvestris*	IAC collection	SjCer6
Cerovica	*Vitis v.subsp. sylvestris*	IAC collection	SjCer7
Cerovica	*Vitis v.subsp. sylvestris*	IAC collection	SjCer8

Cerovica	*Vitis v.subsp. sylvestris*	IAC collection	SjCer9
Cerovica	*Vitis v.subsp. sylvestris*	IAC collection	SjCer10
Cerovica	*Vitis v.subsp. sylvestris*	IAC collection	SjCer11
Cerovica	*Vitis v.subsp. sylvestris*	IAC collection	SjCer12
Cerovica	*Vitis v.subsp. sylvestris*	IAC collection	SjCer13
Cerovica	*Vitis v.subsp. sylvestris*	IAC collection	SjCer14
Cerovica	*Vitis v.subsp. sylvestris*	IAC collection	SjCer15
Cerovica	*Vitis v.subsp. sylvestris*	IAC collection	SjCer16
Cerovica	*Vitis v.subsp. sylvestris*	IAC collection	SjCer17
Cerovica	*Vitis v.subsp. sylvestris*	IAC collection	SjCer18
Cerovica	*Vitis v.subsp. sylvestris*	IAC collection	SjCer19
Cerovica	*Vitis v.subsp. sylvestris*	IAC collection	SjCer20
Cerovica	*Vitis v.subsp. sylvestris*	IAC collection	SjCer21
Cerovica	*Vitis v.subsp. sylvestris*	IAC collection	SjCer22
Cerovica	*Vitis v.subsp. sylvestris*	IAC collection	SjCer23
Cerovica	*Vitis v.subsp. sylvestris*	IAC collection	SjCer24
Cerovica	*Vitis v.subsp. sylvestris*	IAC collection	SjCer25
Cerovica	*Vitis v.subsp. sylvestris*	IAC collection	SjCer26
Cerovica	*Vitis v.subsp. sylvestris*	IAC collection	SjCer27

nd, not determined
